# Prevalence and Symptom Profile of Long COVID among Schoolchildren in Vietnam

**DOI:** 10.3390/v16071021

**Published:** 2024-06-25

**Authors:** Trang Thu Vu, Khanh Cong Nguyen, Hieu Thi Nguyen, Anh Hoang, Nghia Duy Ngu, Duong Nhu Tran, Hoa Bich Phan, Ha Thi Thu Nguyen, Thai Quang Pham, Florian Vogt

**Affiliations:** 1 National Centre for Epidemiology and Population Health, Research School of Population Health, College of Health and Medicine, Australian National University, Canberra, ACT 2601, Australia; florian.vogt@anu.edu.au; 2 Department of Communicable Disease Control, National Institute of Hygiene and Epidemiology, Hanoi 100000, Vietnam; nck@nihe.org.vn (K.C.N.); ndn@nihe.org.vn (N.D.N.); tnd@nihe.org.vn (D.N.T.); pqt@nihe.org.vn (T.Q.P.); 3 Field Epidemiology Training Program, National Institute of Hygiene and Epidemiology, Hanoi 100000, Vietnam; 4 Thai Nguyen Centers for Disease Control and Prevention, Thai Nguyen 250000, Vietnam; daonguyenngocdiep@gmail.com (H.T.N.); anh197@gmail.com (A.H.); thuha2836@gmail.com (H.T.T.N.); 5 Thai Nguyen District Health Center, Thai Nguyen 250000, Vietnam; bichhoatn@gmail.com; 6 Research Methodology and Biostatistics Department, SPMPH, Hanoi Medical University, Hanoi 100000, Vietnam; 7 The Kirby Institute, University of New South Wales, Sydney, NSW 2000, Australia

**Keywords:** long COVID, symptom profile, schoolchildren, SARS-CoV-2

## Abstract

Background: Long COVID is a recognized condition that can follow SARS-CoV-2 infection. It has been primarily observed and studied in adults. Evidence on long COVID among children is scarce. We aimed to estimate its prevalence and symptom profile among schoolchildren, and its effects on studying, daily activities, and quality of life. Methods: We conducted a cross-sectional online survey among caregivers of 2226 schoolchildren aged 12–17 in Thai Nguyen, Vietnam, from 11 April to 16 May 2023 using WHO definitions and a validated quality of life questionnaire. Results: Among 1507 children with confirmed SARS-CoV-2 infection ≥ 5 months prior, 85 (5.6%) had long COVID. Memory loss (85.9%), poor concentration capacity (58.8%), and fatigue (57.6%) were their most common symptoms. They reported more frequent interference with their studies, observed differences in school absence rates, reduced daily activities, worsened overall health status, and relatively higher utilization of health services compared with children who only suffered from acute COVID-19 symptoms after infection. Conclusions: Given the near-ubiquitous exposure to SARS-CoV-2 among children at this stage of the pandemic, our findings contribute invaluable evidence of an emerging public health burden among the pediatric population in Vietnam and globally. Concerted public health measures are needed to reduce long-term impacts on health, education, and wellbeing.

## 1. Introduction

Since the beginning of the pandemic on 21 January 2020 until 16 February 2022, there have been over 490,000 confirmed cases of COVID-19 in children in Vietnam, accounting for 19.2% of all persons under the age of 18. Among those testing positive for the virus, 4.8% were children aged 13–17 years, 8% were children aged 6–12 years, 2.8% were children aged 3–5 years, and 3.6% were children aged 0–2 years [1].

Thai Nguyen, located in the Northeast region of Vietnam, is a midland province and part of the Hanoi Capital Region. The province reported a total of 348.081 confirmed cases of SARS-CoV-2 up to 31 July 2023 [2]. To combat the pandemic, Thai Nguyen Province began vaccinating its entire population against COVID-19, and vaccinations for children aged 12 to 17 years started in November 2021. The target group for vaccination included all children within the age range of 12–17 years who were eligible to receive the vaccine based on the manufacturer’s recommendations and the Ministry of Health, with an expected number of 100,121 children [3]. As of 8 March 2022, the vaccination rate for children aged 12–17 in Thai Nguyen Province was 98.4% for the first dose and 96.9% for the second dose [4].

The age group of 12–17 years old is at a critical stage that requires attention and care from parents to ensure their good health. Early detection of long COVID symptoms will enable parents to react promptly and provide necessary support to enhance the health of their children. 

The term long COVID refers to a set of symptoms and health issues that persist as a result of COVID-19 infection and cannot be explained by any other cause. There is currently not one single, universally accepted definition of long COVID. In this study, we use the World Health Organization (WHO) definition, which defines long COVID as symptoms that appear three months after SARS-CoV-2 infection and last for two months or longer [5]. Long COVID has been identified as an emerging public health issue in the wake of the SARS-CoV-2 pandemic, with children being affected too. The most common symptoms are insomnia, fatigue, anosmia, and headaches, with more than 200 symptoms suggested by the WHO [5]. A comprehensive systematic review of long COVID in children and adolescents globally from June 2022 indicated that the prevalence of long COVID in children and adolescents ranged from 1% to 51%, with studies with smaller sample sizes reporting higher rates [6]. A study from Russia in 2021 which involved 518 children hospitalized due to COVID-19, showed that 10.7% reported experiencing fatigue, and the happiness index scores also decreased among the surveyed children [7]. Additionally, a nationwide survey in the United Kingdom on long COVID showed a current prevalence of 0.16% in children aged 2–11 years, 0.65% in children aged 12–16 years, and 1.22% in individuals aged 17–24 years [8].

Long COVID symptoms may result from a mix of factors related to the COVID pandemic and lockdown, such as social isolation, anxiety, depression, or educational concerns, with or without direct evidence of SARS-CoV-2 infection. Overall, there are significant uncertainties surrounding the clinical presentation, diagnosis, prevalence, duration, and also treatment of long COVID [6,9]. 

In Vietnam, the Ministry of Health has published several announcements on long COVID among children to raise awareness among parents, caregivers, and teachers. However, to our knowledge, no study has yet been conducted to investigate the prevalence and range of symptoms of long COVID among children in Vietnam. Therefore, signs of Long COVID might be misidentified and not appropriately treated, leading to complications for children in Vietnam.

The aim of this study was to describe the prevalence of long COVID and the impact of long COVID on studying and daily activities among schoolchildren aged 12–17 years in Thai Nguyen City, Vietnam. The findings of this research will be valuable in guiding pediatric practitioners, teachers, parents, and health policymakers to provide better care and support for children’s health in Vietnam.

## 2. Materials and Methods

### 2.1. Study Design and Setting

This was a cross-sectional online survey conducted from 11 April to 16 May 2023 among parents/guardians of children enrolled at the two schools with the highest prevalence of COVID-19 in Thai Nguyen City, including one secondary school and one high school. The inclusion criteria for the survey were: (1) being a parent/guardian of schoolchildren aged 12–17 years who were studying at one of these two schools during the study period, and (2) being able to answer the questionnaire. There were a total of 3087 eligible parents/guardians in these two schools (based on the total number of enrolled students). In this study, we used the WHO definition to classify symptoms that appeared three months after SARS-CoV-2 infection and lasted two or more months after a child’s COVID-19 infection as long COVID. Therefore, these symptoms may take up to five months after the children’s infection to develop and persist long enough to meet this definition. As a result, we only reported this condition among schoolchildren who had been infected 5 months prior to the survey date (May 2023) to ensure that enough time had passed to identify long COVID symptoms. 

### 2.2. Procedure and Measurement

In Vietnam, each class usually uses an online group on a popular social platform called Zalo to connect teachers with parents/guardians, allowing teachers to communicate with all parents/guardians of children in their classes quickly and effectively. After obtaining permission from school headmasters and headroom teachers to invite parents/caregivers to complete a short anonymous online survey, we sent a RedCAP online survey to Zalo groups of parents/guardians in the two schools. Headroom teachers and school staff were not involved in the survey response process to avoid potential biases.

### 2.3. Independent Variables

Demographic information collected from the participants included: age, sex (male/female), age, COVID-19 vaccination status (Yes/No/Do not know), number of COVID-19 vaccine doses received, type of COVID-19 vaccine received (Pfizer/Moderna/Other/Do not know), underlying medical conditions (Yes/No/Do not know). 

### 2.4. Outcome Variables

#### 2.4.1. Long COVID

Long COVID includes symptoms that appear three months after SARS-CoV-2 infection and last for two months or longer after a child’s COVID-19 infection [5]. Data on nineteen symptoms were collected by Yes/No questions, including: Cough, Fever, Loss of smell, Shortness of breath, Sore throat, Headache, Muscle pain, Abdominal pain, Diarrhea, Tiredness or fatigue that interferes with daily life, Sleep disorders (cannot sleep, sleep too much), Anxiety/depression, Poor concentration, Memory issues, Dizziness when the child stands up (light-headedness), Sore eyes, Raised welts on skin or swelling ear ache or ringing in the ears, and Red or purple sores or blisters on feet. 

We also collected data on the impact of long COVID on the child’s studying and daily activities. Parents/guardians’ perceptions of their child’s health were surveyed to determine if they believed that the child had fully recovered from COVID-19. The severity of long COVID was reported based on whether the child had visited a doctor, hospital, commune health center, emergency department, or did not attend school due to any long COVID symptoms.

#### 2.4.2. The EQ-5D-Y Questionnaire

The assessment of quality of life utilized the EuroQol-five (EQ-5D-5L) tool [10]. This instrument encompasses five distinct dimensions that capture various facets of an individual’s well-being, specifically focusing on mobility, self-care, regular activities, discomfort/pain, and depression/anxiety. Each dimension employs a Likert rating scale comprising five response levels, with values ranging from ‘no problems’ coded as 1 to ‘slight problems’ coded as 2 and ‘severe problems’ coded as 3. To compute the overall EQ-5D-5L score, the crosswalk value set from the EuroQuoL data (EuroQol Research Foundation, 2019) was used.

The Vietnamese version of EQ-5D-5L has been used and validated in many previous studies conducted in Vietnam [11,12]. However, in this study, we used the shortened version of the EQ-5D-5L, which is the EQ-5D-Y for children, because it has been studied to be shorter and more suitable for the children’s age [13].

### 2.5. Data Analysis

The data were summarized using descriptive statistics, namely frequencies and percentages for categorical variables. The descriptive statistics were calculated using Stata 17 software (Stata Corporation).

We compared students with long COVID against children who had prior SARS-CoV-2 infection and at least one acute symptom, and also against children who had no prior SARS-CoV-2 infection but who also had at least one acute symptom. Acute symptoms were defined as COVID-19 compatible symptoms that children with prior SARS-CoV-2 infection experienced since the last time they were tested positive until 16th May 2023 (survey ended); for those schoolchildren without prior SARS-CoV-2 infection, these were symptoms that children experienced between 10 February 2022 (school opening) until 16th May 2023 (survey ended). 

For the descriptive analysis, first, we compared children without prior infection since school opening on 10 February 2022 and children with prior infection since the last positive COVID-19 test. According to the above definitions, we then compared three groups of students who had at least one acute symptom: children without prior SARS-CoV-2 infection since school opening, but who nevertheless had at least one acute symptom; children with prior SARS-CoV-2 infection and at least one acute symptom; and children with long COVID.

## 3. Results

A total of 2398 parents/guardians opened our survey, and from those, 2339 parents/guardians consented and participated. We excluded 113 completed surveys: 19 due to logical errors of parents/guardians and 94 because students had turned 18 years of age by the time of the survey. In total, survey data from 2226 children were included in our study. 

Table 1 shows the characteristics of schoolchildren in Thai Nguyen City with and without prior SARS-CoV-2 infection. Among infected children, the prevalence of female students was 52.1%, and that among male students was 47.9%. Among those without prior COVID-19 infection, the prevalences of female and male students were 51.6% and 48.4%, respectively. A proportion of 90.4% of non-infected students were vaccinated, and 94.5% of infected students were vaccinated. Most of the schoolchildren had received three doses of the vaccine (60.3% among non-infected and 54.3% among infected), followed by two doses of the vaccine (19.2% among non-infected and 29.2% among infected). Among schoolchildren without prior infection, 67.9% were vaccinated with Pfizer, 8.3% with Moderna, and 4.7% had not been vaccinated. Among those with prior infection, the prevalence was 77.0%, 8.2%, and 4.5% for Pfizer, Moderna, and unvaccinated, respectively. 

Nearly all children had no underlying or chronic medical conditions, with 88.9% among non-infected and 92.3% among those infected with COVID-19.

**Figure 1:**
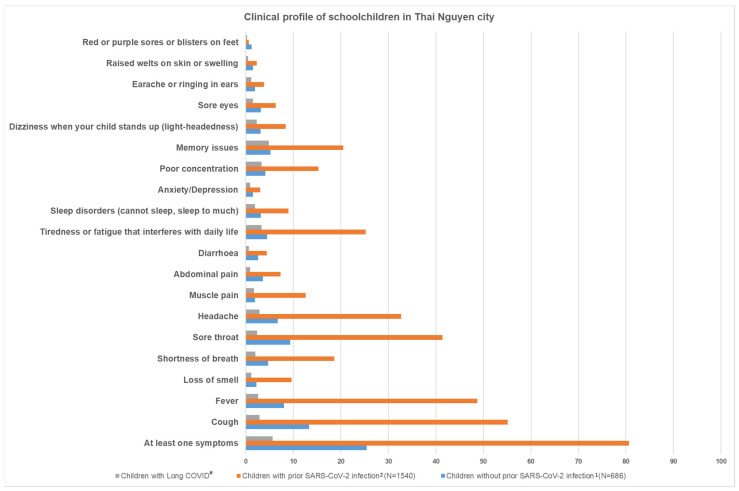
Clinical profile of schoolchildren in Thai Nguyen City. ^#^ among n = 1507 children with SARS-CoV-2 infection ≥ 5 months prior, who experienced at least one long COVID symptom appearing within three months after infection lasting for at least two months. ^1^ since school opening on 10 February 2022. ^2^ since the last positive COVID-19 test.

Figure 1 describes the clinical profile of all schoolchildren in Thai Nguyen City, separately for acute symptoms (with and without prior infection) and for long COVID condition.

Among the 686 schoolchildren without prior infection, 25.4% had at least one acute symptom at the time of the survey; 13.3% had a cough, 9.3% sore throat, 8.0% fever, 6.7% headache, 5.2% memory issues, 4.7% shortness of breath, 4.1% poor concentration, and 3.6% abdominal pain.

Among the 1540 schoolchildren with prior infection, 80.6% had at least one acute symptom: 55.1% had a cough, 48.7% fever, 41.4% sore throat, 32.7% headache, 25.2% felt tired or fatigued that interfered with their daily life, 20.5% memory issues, 18.6% shortness of breath, 15.3% poor concentration, and 12.6% muscle pain.

Among the 1507 schoolchildren who had experienced COVID-19 infection ≥ 5 months prior to the survey date, there were 5.6% with at least one long COVID symptom. 4.8% had memory issues, 3.3% felt tired or fatigued, 2.9% a had cough, 2.9% had a headache, 2.6% had a fever, 2.4% had a sore throat, 2.3% had light-headedness, 2.0% had shortness of breath, and less than 2% had other symptoms (muscle pain, abdominal pain, diarrhea, sleep disorders, anxiety/depression, sore eyes, raised welts on skin or swelling earache or ringing in the ears, or purple–red sores or blisters on feet).

**Figure 2:**
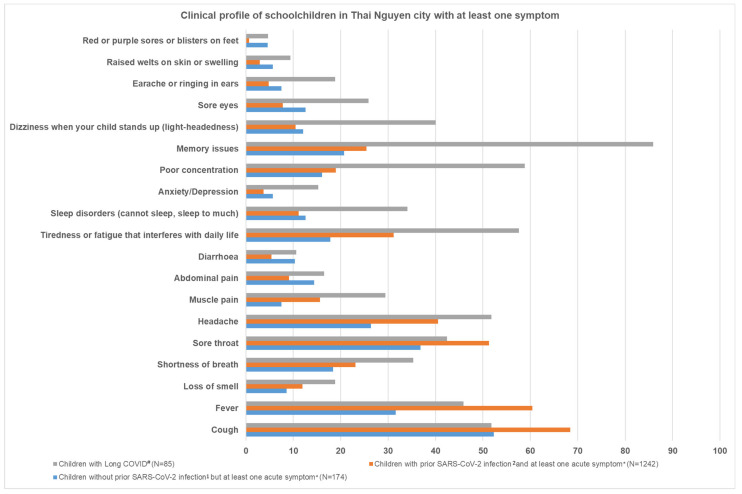
Clinical profile of schoolchildren in Thai Nguyen City with at least one symptom. ^#^ among n = 1507 children with SARS-CoV-2 infection ≥ 5 months prior, who experienced at least one long COVID symptom appearing within three months after infection and lasting for at least two months. * for schoolchildren without SARS-CoV-2 infection: symptoms that schoolchildren experienced since the last time they tested positive until 16 May 2023 (survey end); for schoolchildren without SARS-CoV-2 infection: symptoms that children experienced between 10 February 2022 (school opening) until 16 May 2023 (survey end). ^1^ since school opening on 10 February 2022. ^2^ since the last positive COVID-19 test.

Figure 2 shows the clinical profile of schoolchildren with at least one acute symptom or long COVID in Thai Nguyen City. 

Among 174 children with at least one acute symptom without prior COVID-19 infection, 52.3% had a cough, 36.8% had a sore throat, 31.6% had a fever, 26.4% had a headache, 20.7% had memory issues, 18.4% had shortness of breath, 17.8% had tiredness or fatigue, 16.1% had poor concentration, and less than 15% had other symptoms.

Among 1242 schoolchildren with at least one acute symptom and prior COVID-19 infection, 68.4% had a cough, 60.4% had a fever, 51.3% had a sore throat, 40.5% had a headache, 31.2% had tiredness or fatigue that interferes with daily life, 25.4% had memory issues, 23.1% had shortness of breath, 19% had poor concentration, 15.6% had muscle pain and less than 15% had other acute symptoms.

There were 85 schoolchildren who had at least one long COVID symptom. Among them, 85.9% had memory issues, 58.8% had poor concentration, 57.6% had tiredness or fatigue that interferes with daily life, 51.8% had a cough, 51.8% had a headache, 45.9% had a fever, 42.4% had a sore throat, 40% had dizziness or light-headedness when standing up, 35.3% had shortness of breath, 34.1% had sleep disorders, 29.4% had muscle pain, 25.9% had sore eyes, 18.8% had loss of smell, 18.8% had earache or ringing in ears, 16.5% had abdominal pain, 15.3% had anxiety or depression, 10.6% had diarrhea, 9.4% had swelling or raised welts on skin, and 4.7% had red or purple sores or blisters on their feet.

Table 2 shows the effects of acute symptoms and of long COVID symptoms on studying and daily activities among schoolchildren in Thai Nguyen City with at least one symptom. When parents/guardians were asked whether they thought the child’s studying has been affected by any symptoms, 46% of parents/guardians of children with at least one long COVID symptom somewhat agreed and 16% strongly agreed with this statement. Among parents/guardians of children with at least one acute symptom and with prior infection, 27.6% somewhat agreed and 15.5% strongly agreed. For those with at least one acute symptom but without prior infection, 27.6% somewhat agreed and 9.2% strongly agreed.

A proportion of 18.1% of parents or guardians of children who were infected and displayed at least one acute symptom indicated that their child required more than a month to resume normal daily activities or had to increase their resting hours.

A proportion of 27% of parents/guardians of children who had at least one Long COVID symptom somewhat agreed and 16% strongly agreed that their child had fully recovered. For children with at least one acute symptom, 23.2% somewhat agreed and 25.2% strongly agreed.

Table 3 describes the effects of acute and long COVID symptoms on health care utilization for schoolchildren in Thai Nguyen City with at least one symptom. Among children with at least one acute symptom without prior infection, 10.9% saw a doctor or visited a commune health center/hospital, 2.3% attended an emergency department, 2.3% were admitted to hospital, and 19.5% had to stay at home from school because of clinical symptoms. The prevalences among those with acute symptoms and prior infection were 11.1%, 1.6%, 2.7%, and 29.4%, respectively.

Among schoolchildren who had at least one long COVID symptom, 15% saw a doctor, 5% attended an emergency department, 8% were admitted to hospital, and 36% had to stay at home from school because of any of the long COVID symptoms.

Table 4 shows the health and quality of life of schoolchildren in Thai Nguyen City by EQ-5Y-D dimension and severity level. Most children without prior infection at the time of survey had no problems in any dimension; 1.3% reported some/a lot of problems regarding mobility, 1.3% reported some/a lot of problems with regards to looking after themselves, 0.9% reported some/a lot of problems in performing usual activities, 1.2% reported having some/a lot of pain or discomfort, and 2.9% reported some problems and 0.7% a lot of problems due to feeling worried/sad/unhappy.

Among children with prior infection, the prevalences of children with some or a lot of problems in mobility, looking after themselves, performing their usual activities, having discomfort or pain, and feeling unhappy/sad/worried were 1.2% and 0.1%; 0.9% and 0.5%; 2.0% and 0.3%; 7.2% and 0.4%; and 8.9% and 0.6%, respectively. For the same schoolchildren at the time of the survey, i.e., after infection, the percentages reported for these dimensions were 0.8% and 0.1%; 0.5% and 0.1%; 1.3% and 0.1%; 3.9% and 0.3%; and 6.4% and 0.5%, respectively.

## 4. Discussion

In this study, we investigated schoolchildren aged 12 to 17 years old for acute symptoms with and without prior SARS-CoV-2 infection, long COVID symptoms, and quality of life. We found a prevalence of long COVID in our study of 5.6%. While the majority (about 80%) of children with prior infection showed at least one COVID-19 compatible symptom, about one-quarter without prior infection also showed at least one of these symptoms. Children with prior COVID-19 infection and with at least one long COVID symptom reported a noticeably higher prevalence of visiting a doctor, going to the emergency department, being admitted to a hospital, and having more school absences than in the COVID-19 test-negative group. Surprisingly, in contrast, children who did not have a previous COVID-19 infection demonstrated slightly higher quality of life. 

A total of 2226 schoolchildren were included in our study. There were 686 children without prior COVID-19 infection and 1540 with prior infection. Among schoolchildren who had been infected, there were 1507 infected ≥5 months prior to the surveyed date. The prevalence of at least one acute symptom and acute symptoms among those with prior infection was subtly higher than those without (80.6% versus 25.4%). 

In this study, we used the WHO’s definition of long COVID as a continuation or development of new symptoms three months after infection, and the duration of symptoms lasting two months or longer [5]. The prevalence of long COVID symptoms among those infected was 5.6%, which is lower compared with a meta-analysis of 21 studies (25.4%) and a nation-wide cohort study in Denmark showing 28% among children aged 6–17 [6,14]. Moreover, a cohort study in Germany reported that 7.5–10% of children from 12 to 17 years old had long COVID [15]. This difference may be due to each study’s definition of long COVID, which requires the presence of one or more symptoms lasting for more than four weeks after a SARS-CoV-2 infection, in contrast to our definition and age range [6,14,15]. The prevalence of long-lasting COVID-19 symptoms could also depend on the number, type of symptoms surveyed, and time of follow-up [16,17].

The observed pattern showed lower prevalence of the presence of at least one acute symptom among children without prior infection than those with prior infection (25.4% and 80.6% respectively). This finding aligned with a longitudinal study in the United Kingdom that showed that symptoms were more common in test-positive compared with test-negative children [18].

Among children without prior COVID-19 infection, the five most acute symptoms were cough, sore throat, fever, headache, and memory issues, ranging from 5.2–13.3%. The most common acute symptoms among schoolchildren with prior infection were cough, fever, sore throat, headache, fatigue, memory issues, shortness of breath, poor concentration, and muscle pain, ranging from 12.6–68.4%. The most common long COVID symptoms recorded were memory issues, fatigue, cough, headache, fever, sore throat, light-headedness, and shortness of breath. The prevalence of 19 symptoms ranged from 4.7–85.9%. 

Previous studies have described memory issues, poor concentration, headache, cough, and fever as long COVID symptoms of SARS-CoV-2 infection in children [19,20]. Stephenson et al. found similar symptoms persistent after 3 months among their COVID-19 test-positive group, including tiredness, headache, and shortness of breath, and among the negative group, tiredness and headache [8]. One study from the United Kingdom among school-aged children also reported the most common symptoms as headache, fatigue, fever, and cough, which is similar to our finding [21]. Furthermore, a systematic review of twenty-one studies on long COVID in children and adolescents discovered that the five most common clinical symptoms were fatigue, mood symptoms, sleep disorders, headache, and respiratory symptoms, which also aligns with our findings [6]. A study conducted in England and Wales among children reported differences in clinical symptoms between groups with and without prior infection [22]. In these studies and ours, despite varying definitions of long COVID, the findings consistently highlight the presence of such symptoms, warranting further investigation.

The group of children who presented with at least one symptom of long COVID had a notably higher percentage of visits to a health center or emergency department and hospitalization in comparison with those who had at least one acute symptom, with or without prior COVID-19 infection.

Children with at least one long COVID symptom had the highest rate of school absence, followed by those infected and those uninfected with at least one acute symptom (36% versus 29.4% versus 19.5%). Our findings were consistent with a Danish study, which found that children who were infected with COVID-19 experienced a greater number of sick days and related absences from school [17].

A notable variation in the proportion of parents or guardians (62%) of children who had experienced at least one long COVID indicated that their child’s academic performance had been negatively affected by the presence of any of these symptoms. This percentage was comparatively lower for children who had been infected with COVID-19 and who had at least one acute symptom (43.1%), as well as for children who had not been infected (36.8%). The results of our study indicate that the presence of COVID-19 symptoms, both acute and long COVID, had a tendency to affect the well-being of schoolchildren.

The majority of parents or guardians of infected children indicated that their child was able to resume regular daily activities. However, the prevalence of this ability was greater among those children who had at least one acute symptom, as opposed to those who experienced at least one long COVID symptom (77.3% versus 64%). The prevalence of long COVID symptoms was apparently higher (32%) among individuals who experienced at least one symptom, potentially due to the prolonged duration of these symptoms.

A mere 1.3% of parents or guardians of children presenting with at least one acute symptom indicated that their child experienced limitations in their daily activities or had to discontinue them altogether. Conversely, none of the parents or guardians whose children displayed at least one symptom associated with long COVID reported similar restrictions. The potential cause may be attributed to the magnitude of symptoms exhibited by the children.

When asked about the complete recovery of their children from COVID-19, it was found that 48.4% of parents or guardians of infected children acknowledged the presence of at least one acute symptom, in contrast with 43% of those who exhibited at least one symptom associated with long COVID. The present study provides evidence regarding the effects of long COVID symptoms on the overall well-being of children who contracted COVID-19, which is similar to previous studies that also reported limitations in the daily functioning of children due to long COVID. Research conducted in Sweden found that COVID-19 infection substantially disrupted children’s everyday routines, including decreased school attendance and limited participation in daily activities [23]. Moreover, Ashkenazi-Hoffnung et al. found that a majority of 58.9% out of a sample of 90 children experienced limitations in their daily activities as a result of symptoms that persisted for a duration exceeding four weeks [24]. In a separate study conducted on the Dutch population, it was revealed that 36% of children faced substantial restrictions in their daily functioning due to symptoms that persisted for a duration exceeding twelve weeks [25]. Likewise, a nationwide study conducted in Denmark observed a decrease in social and school functioning among children who had contracted the infection [17]. Despite differences in the definition of long COVID, population, and time, prior research found similar impacts of prolonged COVID-19 symptoms on day-to-day functioning of children as the findings in our study.

We surveyed quality of life among schoolchildren through five dimensions: mobility, looking after themselves, having pain or discomfort, performing usual activities, and feeling unhappy/worried/sad.

At the time of the survey, the prevalence of having problems with mobility and looking after themselves among children without infection was somewhat higher than that among children with prior infection (1.3% versus 0.9%; 1.3% versus 0.6%). For performing usual activities, having discomfort/pain, and feeling unhappy/worried/sad, the prevalence of having problems among children non-infected with COVID-19 was slightly lower than those infected at the time of the survey (0.9% versus 1.4%; 1.2% versus 4.2%; and 3.6% versus 6.9%; respectively).

A national Danish study, which used the Pediatric Quality of Life questionnaire, revealed that children who had contracted COVID-19 exhibited slightly higher physical functioning problems [17]. However, among non-infected children, all other aspects of functioning were relatively lower, possibly due to variations in the tools used to assess children’s quality of life. Furthermore, a study conducted by Borch et al. using the WHO-5 well-being questionnaire [26] found that SARS-CoV-2-positive children reported a noticeably higher sense of well-being than those who had never tested positive; however, the WHO-5 questionnaire only focuses on mental health dimensions [14]. One probable explanation for the disparity is that the demographic and clinical features of these groups are different. It is also possible that the uninfected children were afraid of the unknown diseases and had a more restricted daily life in order to avoid contracting the virus. They also had some acute symptoms, similar to infected children, but their test results were negative, which may have caused anxiety in this group of children. 

It is worth noting that schoolchildren who had contracted COVID-19 exhibited a greater prevalence of issues across all five dimensions prior to their infection compared with the present time of the survey, albeit at low overall levels. Specifically, 1.3% versus 0.9% for mobility, 1.4% versus 0.6% for self-care, 2.3% versus 1.4% for normal activities, 7.6% versus 4.2% for pain or discomfort, and 9.5% versus 6.9% for feeling worried/sad/unhappy. This could be attributed to COVID-19 restrictions and policies of social distancing, which increased childcare time and improved their well-being. In addition, it is possible that parents cared more for their children when the child was sick, which might have resulted in positive effects in those dimensions. 

We also observed that there were symptoms that were reported to be notably difference among children without prior infection who had at least one acute symptom, which were abdominal pain, diarrhea, sleep disorder, anxiety/depression, light-headedness, sore eyes, raised welts on skin or swelling, ear ache or ringing in the ears, or purple–red sores or blisters on feet. Prior research also stated a hypothesis that symptoms with a higher frequency present in the uninfected group could be attributed to children’s well-being [14]. Hence, it is imperative to evaluate whether these symptoms are indicative of the adverse consequences of the social implications of the pandemic on the physical and mental well-being of children.

It is important to acknowledge some important limitations of our study. First, case confirmation did not require PCR-confirmed tests, which could have resulted in some cases being missed and possibly being in the uninfected group of children. Second, our study was conducted within the context of the “new normal”, thereby limiting our ability to examine the impact of COVID-19 implications on the well-being of children. Third, a cross-sectional study design was used, which introduced the potential for bias due to reliance on the recall memory of parents or guardians. The response rates in our study were 68% among parents of secondary school children and 86% among parents of high school children. This indicates that a proportion of 14% to 32% of parents/guardians of schoolchildren chose not to partake in our research, potentially introducing selection bias into our study. In addition, our study sample was selected by convenience, which involved the of local authorities to choose two schools with the highest prevalence of COVID-19 infection. We were not able to estimate the potential presence and extent of such bias and how this might have affected our observed results. Consequently, the outcomes of our study may have limited generalizability to the entire population of parents of schoolchildren in Thai Nguyen Province. Additionally, in our study, parents filled out the quality-of-life part on behalf of the student, entering their responses as reported. This might have led to information bias, as some children may be hesitant to fully communicate their health status with their parents for personal reasons. Finally, this study did not collect information on how long it had been since schoolchildren received their most recent vaccination. Ascertaining a child’s complete vaccination status during their most recent infection was difficult and limited our ability to determine their immunization status with certainty.

## 5. Conclusions

Schoolchildren aged 12 to 17 years old who had previously been infected had a slightly higher prevalence of acute symptoms, and long COVID symptoms were only seen in this group. Memory problems, poor concentration, fatigue, cough, and headache were the top-five long COVID symptoms. Children with long COVID symptoms reported a more severe situation, hospitalization, difficulties with studying and daily activities, and school absence. There was a trend toward higher quality-of-life scores in the group of children with a history of COVID-19 infection than in those who had not previously been infected. It is important for families and schools to be cognizant of these signs in students in order to offer timely assistance. The health sector, including school health, should organize educational workshops on probable symptoms following COVID-19 infection. These sessions should aim to help children and parents to identify symptoms and take proactive measures to seek medical attention when needed.

## Figures and Tables

**Table 1:** Characteristics of schoolchildren in Thai Nguyen City with and without prior COVID-19 infection.

	Without Prior SARS-CoV-2 Infection ^#^ (N = 686)	With Prior SARS-CoV-2 Infection * (N = 1540)
**Sex**		
Male	354 (51.6%)	737 (47.9%)
Female	332 (48.4%)	803 (52.1%)
**Age**		
11	40 (5.8%)	202 (13.1%)
12	43 (6.3%)	223 (14.5%)
13	72 (10.5%)	213 (13.8%)
14	51 (7.4%)	258 (16.8%)
15	131 (19.1%)	247 (16.0%)
16	180 (26.2%)	207 (13.4%)
17	169 (24.6%)	190 (12.3%)
**Schooling**		
Secondary school	221 (32.2%)	968 (62.9%)
High school	465 (67.8%)	572 (37.1%)
**Previous COVID-19 vaccination**		
No	32 (4.7%)	70 (4.5%)
Yes	620 (90.4%)	1455 (94.5%)
Don’t know	34 (5.0%)	15 (1.0%)
**Number of COVID-19 vaccine doses received**		
0	32 (4.7%)	70 (4.5%)
1	9 (1.3%)	107 (6.9%)
2	132 (19.2%)	449 (29.2%)
3	414 (60.3%)	836 (54.3%)
4	24 (3.5%)	39 (2.5%)
missing	75 (10.9%)	39 (2.5%)
**Type of COVID-19 vaccine received**		
None	32 (4.7%)	70 (4.5%)
Pfizer	466 (67.9%)	1186 (77.0%)
Moderna	57 (8.3%)	125 (8.1%)
Other	5 (0.7%)	10 (0.6%)
Do not know	95 (13.8%)	135 (8.8%)
Missing	31 (4.5%)	14 (0.9%)
**Underlying/chronic medical conditions**		
No	610 (88.9%)	1421 (92.3%)
Yes	12 (1.7%)	52 (3.4%)
Don’t know	64 (9.3%)	67 (4.4%)
**Children with Long COVID ^1^**		
No	NA	1422 (94.4%)
Yes	NA	85 (5.6%)
**Children with at least one acute symptom ^2^**		
No	512 (74.6%)	298 (19.4%)
Yes	174 (25.4%)	1242 (80.6%)

^1^ among n = 1507 children with SARS-CoV-2 infection ≥ 5 months prior, who experience at least one long COVID symptom appearing within three months after infection lasting for at least two months. ^2^ for schoolchildren without SARS-CoV-2 infection: symptoms that schoolchildren experienced since the last time they were tested positive until 16 May 2023 (survey end); for schoolchildren without SARS-CoV-2 infection: symptoms that children experienced between 10 February 2022 (school opening) until 16 May 2023 (survey end). ^#^ since school opening on 10 February 2022. * since the last positive COVID-19 test. NA: not applicable.

**Table 2:** Effects on studying and daily activities among schoolchildren in Thai Nguyen City with at least one symptom.

	Children without Prior SARS-CoV-2 Infection ^1^ but at Least One Acute Symptom * (N = 174)	Children with Prior SARS-CoV-2 Infection ^2^ and at Least One Acute Symptom * (N = 1242)	Children with at Least one Long COVID ^#^ Symptom (N = 85)
**Your child’s studying has been affected by any of the symptoms**			
Strongly disagree	4 (2.3%)	50 (4.0%)	9 (11%)
Somewhat disagree	42 (24.1%)	242 (19.5%)	6 (7%)
Neither disagree nor agree	28 (16.1%)	199 (16.0%)	12 (14%)
Somewhat agree	48 (27.6%)	343 (27.6%)	39 (46%)
Strongly agree	16 (9.2%)	193 (15.5%)	14 (16%)
Do not know	36 (20.7%)	215 (17.3%)	5 (6%)
Missing	0 (0.0%)	0 (0.0%)	
**Your child has been able to continue his/her usual daily activities?**			
Yes, my child was able to fully return to usual activities of daily life within a month of his/her COVID illness	NA	960 (77.3%)	54 (64%)
Yes, but my child needed more than a month before he/she was able to fully return to usual activities of daily life	NA	157 (12.6%)	12 (14%)
Yes, but my child had to increase rest hours of his/her usual daily life activities	NA	68 (5.5%)	15 (18%)
No, my child had to stop one or many activities of his/her daily life activities	NA	16 (1.3%)	0 (0.0%)
Do not know	NA	41 (3.3%)	4 (5%)
Missing	NA	0 (0.0%)	0 (0.0%)
**As of today, do you think your child has fully recovered from COVID-19**			
Strongly disagree	NA	90 (7.2%)	5 (6%)
Somewhat disagree	NA	121 (9.7%)	15 (18%)
Neither disagree nor agree	NA	145 (11.7%)	17 (20%)
Somewhat agree	NA	288 (23.2%)	23 (27%)
Strongly agree	NA	313 (25.2%)	14 (16%)
Do not know	NA	285 (22.9%)	11 (13%)

^#^ among n = 1507 children with SARS-CoV-2 infection ≥ 5 months prior, who experienced at least one long COVID symptom appearing within three months after infection lasting for at least two months. * for schoolchildren without SARS-CoV-2 infection: symptoms that schoolchildren experienced since the last time they were tested positive until 16 May 2023 (survey end); for schoolchildren without SARS-CoV-2 infection: symptoms that children experienced between 10 February 2022 (school opening) until 16 May 2023 (survey end). ^1^ since school opening on 10 February 2022. ^2^ since the last positive COVID-19 test. NA: not applicable.

**Table 3:** Effects on health care utilization among schoolchildren in Thai Nguyen City with at least one symptom.

	Children without Prior SARS-CoV-2 Infection ^1^ but at Least One Acute Symptom * (N = 174)	Children with Prior SARS-CoV-2 Infection ^2^ and at Least One Acute Symptom * (N = 1242)	Children with Long COVID ^#^ (N = 85)
**Saw a doctor or visited a commune health center/hospital because of any clinical symptoms**			
No	137 (78.7%)	1080 (87.0%)	70 (82%)
Yes	19 (10.9%)	138 (11.1%)	13 (15%)
Do not know	18 (10.3%)	24 (1.9%)	2 (2%)
Missing	0 (0.0%)	0 (0.0%)	0 (0.0%)
**Attended an emergency department because of any of the clinical symptoms**			
No	154 (88.5%)	1202 (96.8%)	79 (93%)
Yes	4 (2.3%)	20 (1.6%)	4 (5%)
Do not know	16 (9.2%)	20 (1.6%)	2 (2%)
Missing	0 (0.0%)	0 (0.0%)	
**Admitted to a hospital because of any of the clinical symptoms**			
No	152 (87.4%)	1193 (96.1%)	77 (91%)
Yes	4 (2.3%)	33 (2.7%)	7 (8%)
Do not know	18 (10.3%)	16 (1.3%)	1 (1%)
Missing	0 (0.0%)	0 (0.0%)	0 (0.0%)
**Had to stay at home from school because of any of the clinical symptoms**			
No	125 (71.8%)	857 (69.0%)	52 (61%)
Yes	34 (19.5%)	365 (29.4%)	31 (36%)
Do not know	15 (8.6%)	20 (1.6%)	2 (2%)
Missing	0 (0.0%)	0 (0.0%)	0 (0.0%)

^#^ among n = 1507 children with SARS-CoV-2 infection ≥ 5 months prior, who experienced at least one long COVID symptom appearing within three months after infection lasting for at least two months. * for schoolchildren without SARS-CoV-2 infection: symptoms that schoolchildren experienced since the last time they tested positive until 16 May 2023 (survey end); for schoolchildren without SARS-CoV-2 infection: symptoms that children experienced between 10 February 2022 (school opening) until 16 May 2023 (survey end). ^1^ since school opening on 10 February 2022. ^2^ since the last positive COVID-19 test.

**Table 4:** Health and quality of life of schoolchildren in Thai Nguyen City by dimension and severity level.

	Children without Prior SARS-CoV-2 Infection ^1^ (N = 686): Status at Time of Survey (N = 686)	Children with Prior SARS-CoV-2 Infection ^2^ (N = 1540)	
EQ-5D-Y Dimension		Status before Their Infection	Status at Time of Survey
**Mobility**			
No problems	677 (98.7%)	1521 (98.8%)	1527 (99.2%)
Some problems	5 (0.7%)	18 (1.2%)	12 (0.8%)
A lot of problems	4 (0.6%)	1 (0.1%)	1 (0.1%)
**Looking After Myself**			
No problems	677 (98.7%)	1519 (98.6%)	1531 (99.4%)
Some problems	2 (0.3%)	14 (0.9%)	7 (0.5%)
A lot of problems	7 (1.0%)	7 (0.5%)	2 (0.1%)
**Doing Usual Activities**			
No problems	680 (99.1%)	1505 (97.7%)	1518 (98.6%)
Some problems	4 (0.6%)	31 (2.0%)	20 (1.3%)
A lot of problems	2 (0.3%)	4 (0.3%)	2 (0.1%)
**Having Pain or Discomfort**			
No problems	678 (98.8%)	1423 (92.4%)	1476 (95.8%)
Some problems	6 (0.9%)	111 (7.2%)	60 (3.9%)
A lot of problems	2 (0.3%)	6 (0.4%)	4 (0.3%)
**Feeling Worried, Sad, or Unhappy**			
No problems	661 (96.4%)	1394 (90.5%)	1434 (93.1%)
Some problems	20 (2.9%)	137 (8.9%)	98 (6.4%)
A lot of problems	5 (0.7%)	9 (0.6%)	8 (0.5%)

^1^ since school opening on 10 February 2022. ^2^ since the last positive COVID-19 test.

## Data Availability

Data is unavailable due to privacy or ethical restrictions.
